# THE CURIOUS CASE OF CONSCIENTIOUSNESS AND DEPRESSION: FINDINGS FROM THE IOWA UNMARRIED SURVIVORS STUDY

**DOI:** 10.1093/geroni/igad104.2587

**Published:** 2023-12-21

**Authors:** Robert Hensley, Alexander Bishop, Peter Martin

**Affiliations:** Southeast Community College, Lincoln, Nebraska, United States; Oklahoma State University, Stillwater, Oklahoma, United States; Iowa State University, Ames, Iowa, United States

## Abstract

The purpose of this study was to analyze the association between personality traits, coping, social support, stress, and loneliness relative to depression reported by N = 227 (M = 78.2, SD = 8.1) participants from the Iowa Unmarried Survivors Study. Using the Development Adaptation Model as a conceptual framework, a series of blocked multiple regression analyses were conducted. In the first block, age, gender, ethnicity, total years of education, and current marital status were included. The second block contained total life events and childhood poverty, whereas the third block included activities of daily living and total number of illnesses. The fourth block contained the aforementioned personality variables, and the fifth block included coping, social support, stress, and loneliness. Lifetime illnesses emerged as a significant predictor of depression, β = .158, p < .01. The more illnesses over one’s lifetime, the higher the levels of depression. In addition, both Neuroticism and Conscientiousness were significant predictors of depression, β = .453, p < .00, β = .139, p < .05 respectively. Finally, stress served as a significant predictor of depression, β = .200, p < .01. This model explained 58% of the variance in depression scores. Being unmarried and navigating through late adulthood presents a challenge to sustaining positive mental health. Results from this study have implications relative to how clinically trained counselors, geriatric social workers, and aging service providers develop interventions and programming to improve mental health and quality of life for persons who remain unmarried throughout later life.

